# A Novel Cassette Method for Probe Evaluation in the Designed Biochips

**DOI:** 10.1371/journal.pone.0098596

**Published:** 2014-06-04

**Authors:** Vitaly Zinkevich, Nelly Sapojnikova, Julian Mitchell, Tamar Kartvelishvili, Nino Asatiani, Samia Alkhalil, Irina Bogdarina, Abdulmohsen A. Al-Humam

**Affiliations:** 1 School of Pharmacy and Biomedical Sciences, University of Portsmouth, Portsmouth, United Kingdom; 2 Andronikashvili Institute of Physics, I. Javakhishvili Tbilisi State University, Tbilisi, Georgia; 3 School of Biological Sciences, University of Portsmouth, Portsmouth, United Kingdom; 4 Saudi Aramco's Research and Development Center, Dharan, Saudi Arabia; University of North Carolina at Charlotte, United States of America

## Abstract

A critical step in biochip design is the selection of probes with identical hybridisation characteristics. In this article we describe a novel method for evaluating DNA hybridisation probes, allowing the fine-tuning of biochips, that uses cassettes with multiple probes. Each cassette contains probes in equimolar proportions so that their hybridisation performance can be assessed in a single reaction. The model used to demonstrate this method was a series of probes developed to detect TORCH pathogens. DNA probes were designed for *Toxoplasma gondii, Chlamidia trachomatis, Rubella, Cytomegalovirus*, and *Herpes virus* and these were used to construct the DNA cassettes. Five cassettes were constructed to detect TORCH pathogens using a variety of genes coding for membrane proteins, viral matrix protein, an early expressed viral protein, viral DNA polymerase and the repetitive gene B1 of *Toxoplasma gondii*. All of these probes, except that for the B1 gene, exhibited similar profiles under the same hybridisation conditions. The failure of the B1 gene probe to hybridise was not due to a position effect, and this indicated that the probe was unsuitable for inclusion in the biochip. The redesigned probe for the B1 gene exhibited identical hybridisation properties to the other probes, suitable for inclusion in a biochip.

## Introduction

DNA microarray technology has been widely used in gene expression analysis, environmental monitoring, and disease characterization. In particular microarrays have allowed the detection of mutations in specific genes that are recognized as diagnostic “markers” for the onset of particular diseases (p53 in GeneChip; HIV in GeneChip, Affimetrix) [1]. In addition, low-density biochips have been developed and used successfully to check the identity of pathogens when detecting disease origins. Examples of this include a biochip for monitoring six sexually transmitted pathogens [2], and one that specifically detects point mutations in the *gyrA* and *parC* genes of *Neisseria gonorrhoeae* that are involved in the formation of ciprofloxacin resistance [3]. A multi-target biochip is ideal for detecting a set of pathogens that cause similar symptoms and co-infections, or that are harmful to the same extent, whether they are encountered singularly or all together [4], [5]. Evaluating multi-target probes can be time consuming, necessitating the need to develop a way of processing a large number of probes simultaneously.

The rate-limiting step in the establishment of a diagnostic biochip is the evaluation of its probes. These probes have to satisfy several requirements such as a uniform melting temperatures (Tm) and being a similar size so that their signal intensities are comparable under the same hybridization conditions. The theoretical profiles of probes, however, are often inconsistent with experimental data, and this can generate biochips with irregular signal responses. It has been estimated that between 21–34% of probes do not match their intended targets [6]. The consequence of this is often the redesign of biochips, costing time before the microarray is fully effective. Using a probe cassette, we have developed a system for testing multiple probes simultaneously so that those with inconsistent hybridization behaviours can be easily identified.

The cassette approach we adopted for probe evaluation allowed multiple sets of oligonucleotides for different genes to be assessed simultaneously under standard hybridization conditions. The cassettes comprise a tandem arrangement of probes, which act as the target DNA, permitting copy number control in standard reactions, so that poorly performing probes can be rapidly identified. As the model for fine-tuning the binding affinity of probes to their target DNA we have used a biochip designed for the identification of 5 pathogens involved in TORCH infections (*Toxoplasma gondii, Chlamydia trachomatis, Rubella, Cytomegalovirus, Herpes virus*). The matrix used for the biochip was a three-dimensional one suitable for depositing a large number of probes [7].

In order for the application of biochips to become routine, flexible and reliable methods that allow probe design and evaluation need to be developed. In this paper we describe a simple and reliable method to evaluate DNA probes using a cassette approach that is applicable to different types of biochips and microarray.

## Materials and Methods

### Probe design and biochip preparation

*De novo* probes were designed for the following targets: the B1 gene of *Toxoplasma gondii*; the major outer membrane protein (MOMP) gene and a cryptic plasmid sequence of *Chlamydia trachomatis*; the envelope protein E1 gene of *Rubella virus (RV)*; the immediate early gene (IE) and the phosphorylated matrix protein pp65 gene of human *Cytomegalovirus*; and the DNA polymerase gene of the *Herpes Simplex Viruses* type 2 (HSV-2). The characteristics of probes are shown in the Table 1. Each probe was modified by the inclusion of 3-methyluridine at the 3′-end (Bioneer Co., Daejeon, Korea) and activated by treatment with NaIO_4_, producing dialdehyde groups that couple with hydrazine groups in the gel matrix [8]. All oligonucleotides were checked for their ability to form secondary and tertiary structures using the IDT Oligo Analyzer programme. Probes were chosen from those ologonucleotides that had similar hairpin formation properties above and below the hybridization temperature.

**Table 1:** Characteristics of the probes used in this study.

Probe name	Target Species	Gene name	GenBank accession no	Sequence 5′→3′	T_m_ °C	G+C %
**CT1**	*Chlamydia trachomatis*	MOMP	X55700	GGA GAT AAT GAG AAC CAT GCT AC	59.1	43.5
**CT2**	*Chlamydia trachomatis*	MOMP	X55700	TCT ATG GGA AGG TTT CGG C	60.0	52.6
**CT3**	*Chlamydia trachomatis*	Cryptic plasmid	X07547	TCA CCA CCT ACA CGG AAA CA	60.0	50
**TOXO1**	*Toxoplasma gondii*	B1	AF179871	CCG GAA ATA GAA AGC CAT G	60.0	47.5
**TOXO2**	*Toxoplasma gondii*	B1	AF179871	GTA TTC GCA GAT TGG TCG C	59.2	52.6
**TOXO3**	*Toxoplasma gondii*	B1	AF179871	GGT GAC GAA AGG GGA AGA AT	60.3	50.0
**TOXO4**	*Toxoplasma gondii*	B1	AF179871	CCT GTT TCC TCT CTT CAC TGT C	58.0	50.0
**CMV1**	*Cytomegalovirus*	IE	M11630 K01090	ATA AGC GGG AGA TGT GGA TG	59.9	50.0
**CMV2**	*Cytomegalovirus*	pp65	M15120	GCG GTG TGC TTT ATT AGG G	58.3	52.6
**HSV**	*Herpes simplex virus*	DNA polymerase	M16321	TTA TCA ACC GCA CCT CCA G	58.6	52.6
**RV**	*Rubela virus*	E1	JX047998	AAC GCC ATT CCC CTG ACT	60.5	50.0
**DBAC**	*Desulfobacter spp.*	16S rRNA	Y14745	GAT AAT CTG CCT TCA AGC CTG G	60.0	50.0

The biochip (0.6×0.6 cm) consisted of 5×5 circular polyacrylamide gel pads, 0.8 mm×0.04 mm, separated by 0.7 mm and fixed on a glass plate. The matrix comprised 9 mM N,N-dimethylacrylamide, 1 mM 2-hydroxyethylacrylate and 0.2 mM N,N methylene–bis-acrylamide, TEMED, glycerol and methylene blue, which was polymerized after UV light treatment and activated by hydrazine hydrate treatment [8]. Activated oligonucleotides (250 pmol) were coupled with the gel pads and two replicate spots of each probe applied to the biochip. The matrix was covered in oil (Immersion oil microscopy grade, AppliChem, Germany) and incubated at 20-25°C for 48 hrs.

### Cassette construction

Five cassettes were synthesized to evaluate the different probes (see Data S1). Cassette N1 (105 bp) contained three probe sequences for *Chlamydia trachomatis*; two for the major outer membrane protein (CT1 and CT2) and one for the cryptic plasmid (CT3). Cassette N2 (139 bp) had the probe sequences for the immediate early protein (CMV1) and the phosphorylated matrix protein pp65 (CMV2) of *Cytomegalovirus*, DNA polymerase of *Herpes simplex virus* type 2 (HSV), the B1 gene of *Toxoplasma gondii* (TOXO1), and the envelope protein of *Rubella virus* (RV). Cassette N3 had the same probe sequences as cassette N2 but in a different order. Cassette N4 (125 bp) contained four different probe sequences for gene B1 of *Toxoplasma gondii* (TOXO1, TOXO2, TOXO3 and TOXO4). Cassette N5 (141 bp) contained five probes (CT1, CMV2, HSV, RV, TOXO2) for detection of each pathogen. All ssDNA cassettes contained the sequencing T7 primer at 5′-end (sense orientation) and the sequencing SP6 primer at 3′-end (antisense orientation).

### PCR amplification of cassettes

The cassettes were amplified from ssDNA using forward T7 (5′-GTA ATA CGA CTC ACT ATA GGG-3′) and reverse SP6 (5′-TAC GAT TTA GGT GAC ACT ATA G-3′) primers. The SP6 primer was modified by the attachment of a fluorescent dye, Cy3, to its 5′ ends, for detection purposes. The high-fidelity DNA polymerase (iProof High-Fidelity DNA polymerase, Bio-Rad) was used to amplify all cassettes. The reactions were initiated by denaturation at 98°C for 30 seconds followed by 35 cycles of 10 seconds at 98°C, 20 seconds at 55°C, 10 seconds at 72°C and a final extension for 10 minutes at 72°C.

### Hybridization and analysis

**Figure 1:**
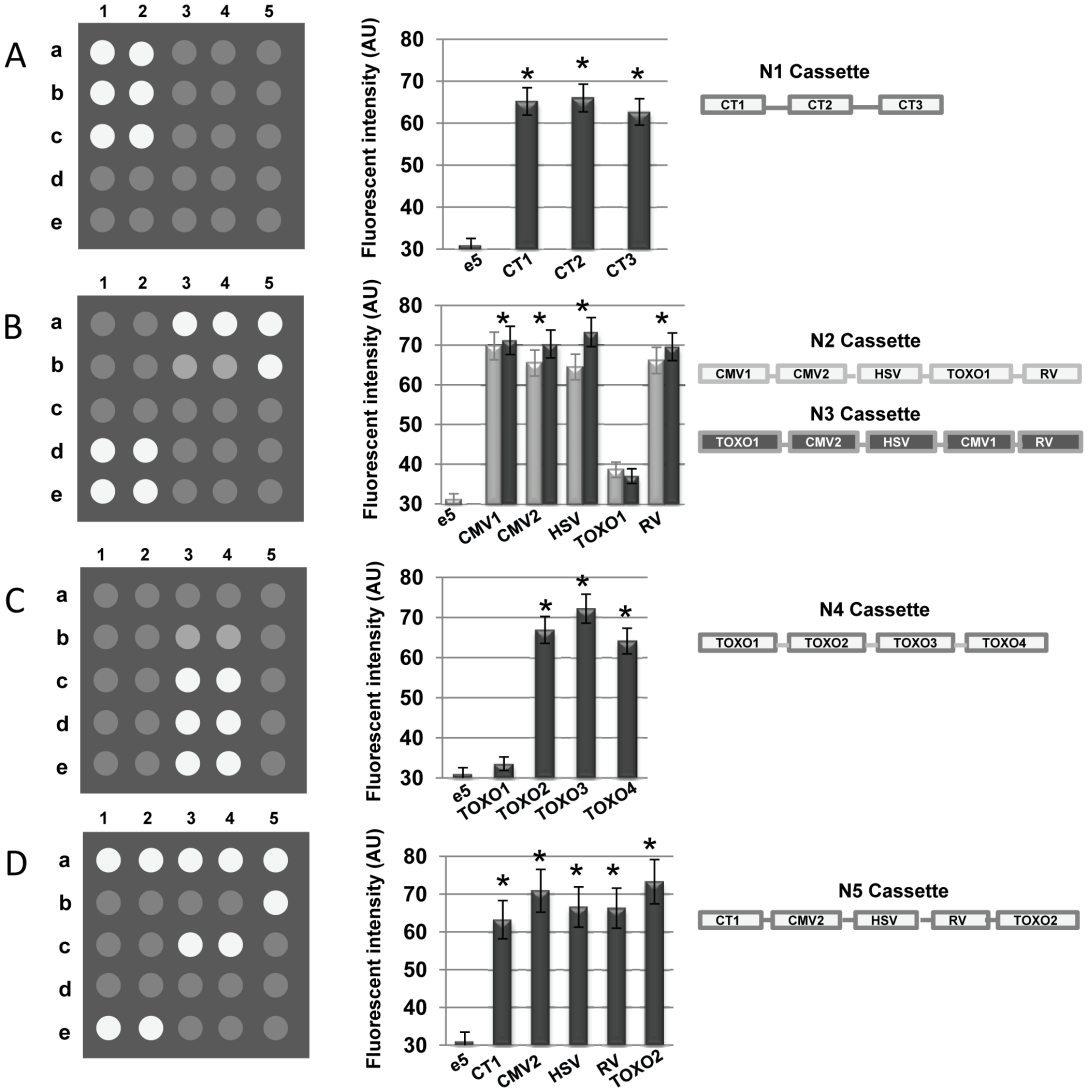
Schematic diagrams and quantitative analysis of the designed cassettes hybridization with biochip. The arrangement of the probes on the biochips (panels A, B, C, and D) was as follows: a1, a2 – CT1; a3, a4 – HSV; a5 – RV; b1, b2 – CT2; b3, b4 – TOXO1; b5 – RV; c1, c2 – CT3; c3, c4 – TOXO2; d1, d2 – CMV1; d3, d4 – TOXO3; e1, e2 – CMV2; e3, e4 –TOXO4; e5 – bacterial DNA (negative control); c5, d5 were left empty to give background signals. White circles represent gene probes that hybridized with the respective targets on the cassette; grey circles represent gene probes that showed no hybridization and background. Error bars in the histograms are from three replicates and present the results of the Student's *t*-Tests procedure (*p<0.05). Panel (A) Cassette N1 shows the hybridization signals for three *Chlamydia trachomatis* probes (probes CT1 and CT2), and for a cryptic plasmid sequence (probe CT3). Panel (B) shows the hybridization signals for the probes CMV1 and CMV2 for *Cytomegalovirus*, the probe HSV for *Herpes simplex virus* (HSV), the TOXO1 probe for *Toxoplasma gondii* (TOXO1), and the RV probe for *Rubela virus* (RV) with cassettes N2 (shaded grey in the histogram) and cassette N3 (shaded black in the histogram). Panel (C) shows the hybridization patterns for the probes TOXO1, TOXO2, TOXO3 and TOXO4 for *Toxoplasma gondii* with cassette N4, revealing that the TOXO1 probe was unsuitable for inclusion in the TORCH biochip. Panel (D) shows the hybridization signals for probes CT1, CMV2, HSV, RV, and TOXO2 with cassette N5.

The probes were evaluated by hybridizing them against oligonucleotide cassettes containing the probe sequences. The cassettes were amplified using PCR and the purified products (QIAquick PCR Purification Kit, Qiagen) were suspended in the hybridization buffer (1 M GuSCN, 50 mM HEPES [pH 7.5], 5 mM EDTA) at a concentration of 100 pmol, heated at 95°C for 5 minutes then quickly quenched on ice and hybridized against the biochip at 45°C in the presence of 0.2 mg/ml bovine serum albumin for 3 hours. The biochips were washed twice with 2X SSC +0.2% SDS at 25°C for 5 minutes, followed by a single wash in 2X SSC at 25°C for 1 minute. The fluorescent patterns were recorded using a Portable Imager 5000 (Aurora Photonics, USA). The negative controls for all hybridization reactions were those probes present on the biochip that were not being evaluated because they were absent in the cassette. In addition to this, DNA from *Desulfobacter spp*. was used as the negative control (Figure 1 e5). Under these circumstances, no cross-hybridisation of the probes was detected. The fluorescent signal intensity of gel elements without immobilized probes was estimated before and after hybridization and used to calculate the median background signal value (Figure 1 c5, d5). The signal intensity of each gel element on the biomatrix was calculated with MicroChip Imager software (Aurora Photonics, USA).

## Results and Discussion

Five cassettes (N1 to N5) were used to evaluate the hybridization potential of each probe. The number of probes included in each cassette ranged from 3 to 5, and the size varied from 105 to 141 bp. The evaluating power of the system can be increased by synthesizing cassettes with a greater number of probes of variable size. Hybridization reactions with the activated biochip were performed under conditions where target DNA and oligonucleotide concentrations were similar, so that the influence concentration on the reaction was negliable. The hybridization behaviours of the *Chlamydia trachomatis* probes were assessed using cassettes N1. All of the probes exhibited comparable hybridization capacities (Figure 1A), indicating that each of these probes were suitable for inclusion in the biochip.

The probes for the *Cytomegalovirus* genes coding for the immediate early protein (CMV1) and the matrix protein pp65 (CMV2), the DNA polymerase of *Herpes simplex virus* (HSV), the B1 protein of *Toxoplasma gondii* (TOXO1), and the envelope protein E1 of *Rubela virus* (RV) were evaluated using cassettes N2 (Figure 1B). Four probes (CMV1, CMV2, HSV and RV) exhibited similar hybridization profiles, whereas the fifth probe (TOXO1) failed to detect its antisense strand. The failed probe was situated at the forth position in cassette N2. To ascertain whether the position of the probe in the cassette influenced its hybridization capacity, we designed cassette N3, where the failed probe was located at the beginning of the cassette. The hybridization behaviour of the N3 cassette did not differ from cassette N2 (Figure 1B), indicating that its hybridization signal was independent of sequence position in the cassette. Probe TOXO1 did, however, hybridize with its single-stranded compliment labeled with a fluorescent dye, Cy3, at the 5′- end (data not shown). This suggested that this probe for the B1 gene needed to be redesigned.

We designed three different probes for the B1 gene from *Toxoplasma gondii*, and synthesized a new cassette, N4 containing all of the probes including TOXO1. The hybridization pattern of cassette N4 showed that probe TOXO1 failed to produce a signal again, confirming that it was not suitable for inclusion in the biochip (Figure 1C). All of the other N4 probes had similar hybridization profiles, any one of which could replace the failed probe. Although all of these probes had similar properties, satisfying the best physico-chemical conditions for hybridization, they exhibited different efficiencies, which were revealed using our cassette evaluation system.

The construction of cassette N5 completed the optimization of this system. This cassette contained a redesigned probe for gene B1 (TOXO2) with the other probes. Each of these probes behaved in a similar fashion when hybridized to their target under the same hybridization conditions (Figure 1D).

In this short communication we describe a practical, emprical approach to the evaluation of probes, ensuring that they share hybridisation characterisitcs so that they can be included in a biochip. The main advantage of the approach is the ability to distinguish the hybridisation capacities of different probe sequences present in equimolar proportions in one long DNA fragment. The method was assessed using five variants, hybridised under the same conditions. Variation in hybridisation intensity is to be expected if the probes have different denaturation/reassociation kinetics. Variation in kinetics behaviour is defined by many factors, including the concentration of the target DNA and the immobilized oligonucleotides, duplex length, G+C content and secondary and tertiary structures in DNA and oligonucleotides [9]–[12]. The concentration of the target DNA, the immobilized oligonucleotides, and the Tm were equivalent for all probes tested. The influence of the secondary and tertiary structures in oligonucleotides was reduced by choosing probes that had similar characteristics for hairpin formation.

DNA microarray development comprises two fundamental areas: the identification of suitable target probes and the determination of optimal hybridization conditions. The cassette approach to probe screening, described in this paper, decreases the time needed to evaluate individual oligonucleotides because it allows them to be processed as a batch in a single reaction. This saving is not insignificant. It can take weeks, or longer, to evaluate and fine-tune the selection of a series of probes for inclusion in a biochip, whereas with our approach it takes hours, once the cassettes have been constructed and prepared. The development time of any biochip can be considerably shortened using this strategy, making it more comparable with that for other multiplex detection systems and other techniques such as probe free nucleic acid detection using isothermal amplification.

The isothermal amplification techniques, which include Loop-Mediated Isothermal Amplification (LAMP) [13], [14], Helicase-Dependent Amplification (HDA) [15], Recombinase Polymerase Amplification (RPA) [16], Rolling Circle Amplification (RCA) [17], are important innovations in the development of clinical diagnosis systems, because they can deliver a qualitative positive or negative response to pathogen detection, particularly when the target is in low amounts, within a short period of time (circa 1–2 hour). Of these LAMP and HDA have been developed commercially [18]. The development of LAMP techniques involve the design and evaluation of complex primer sets, which limited its application initially until software was available for the specific design of these primers [19], [20]. Nevertheless, a series of systems have been designed to detect a range of viral, bacterial and fungal pathogens using LAMP since then [21], however these cannot detect multiple targets in a single reaction. The reasons for this include (1) the use of optical detection, either turbidity or fluorescence, limits the number of signals that can be observed (2) interaction between the additional primer pairs [22], and (3) amplification bias, masking low abundant targets [18]. The development times for biochips and many isothermal amplification techniques cannot be readily compared because one is designed to routinely detect multiple targets in a single reaction, whereas the other is not. In this respect, reducing the time spent designing and evaluating probes is an important contribution to the development of hybridisation biochips.

The detection of low abundant targets, important for clinical sample screening, presents a specific problem for microarrays in that pre-amplification steps (gene specific or whole genome) are necessary before hybridisation. This increases the time needed for target detection and opens the process to potential contamination issues. Furthermore, the amplification stages are reliant on PCR techniques with a requirement for a thermal cycling [23]. Isothermal amplification techniques provide an alternative to PCR to solve the thermal cycling issue [24], however the current range of enzymes available for use have slow processivity, often with methods comprising multiple steps that would lead to complex and expensive microfluidic consumables [23]. At present no isothermal amplification technique has been developed for all TORCH infections, or to amplify multiple targets in a single reaction that would be suitable for inclusion in a hybridisation microarray. A multiplex PCR reaction for TORCH infections has been described [25], and in this paper we describe a series of hybridisation probes for a biochip that have been designed and tested in a relatively short period.

## Supporting Information

Data S1 The composition and sequences of N1, N2, N3, N4 and N5 cassettes.
(DOC)

## References

[1] Dalma-Weiszhausz DD, Warrington J, Tanimoto EY, Miyada CG (2006) The Affymetrix GeneChip Platform: An overview. Methods Enzymol 410: 3–28 10.1016/S0076-6879(06)10001-4 16938544

[2] Yoon HK, Kim JS, Chung IH, Lee SY, Han JR, et al (2010) An oligonucleotide microarray to detect pathogens causing a sexually transmitted disease. BioChip J 4: 105–109 10.1007/s13206-010-4203-z

[3] Zhou W, Du W, Cao H, Zhao J, Yang S, et al (2004) Detection of *gyrA* and *parC* mutations associated with ciprofloxacin resistance in *Neisseria gonorrhoeae* by use of oligonucleotide biochip technology. J Clinical Microbiol 42: 5819–5824 10.1128/JCM.42.12.5819-5824.2004 15583317 PMC535257

[4] Miller MB, Tang Y-W (2009) Basic concepts of microarrays and potential applications in clinical microbiology. Clin Microbiol Rev 22: 611–633 10.1128/CMR.00019-09 19822891 PMC2772365

[5] Arron ST, Skewes-Cox P, Do PH, Dybbro E, Da Costa M, et al. (2011) Validation of a Diagnostic Microarray for Human Papillomavirus: Coverage of 102 Genotypes. J Nucleic Acids Volume 2011, Article ID 756905, 6 pages. doi:10.4061/2011/756905. 10.4061/2011/756905 PMC3139178 21785699

[6] Hager J (2006) Making and using spotted DNA microarrays in an academic core laboratory. Methods Enzymol 410: 135–168 10.1016/S0076-6879(06)10007-5 16938550

[7] Timofeev EN, Kochetkova SV, Mirzabekov AD, Florentiev VL (1996) Regioselective immobilization of short oligonucleotides to acrylic copolymer gels. Nucleic Acids Res 24: 3142–3148 10.1093/nar/24.16.3142 8774893 PMC146065

[8] Guschin D, Yershov G, Zaslavsky A, Gemmell A, Shick V, et al (1997) Manual manufacturing of oligonucleotide, DNA, and protein microchips. Anal Biochem 250: 203–211 10.1006/abio.1997.2209 9245440

[9] Livshits MA, Mirzabekov A (1996) Theoretical analysis of the kinetics of DNA hybridization with gel-immobilized oligonucleotides. Biophysical J 71: 2795–2801 10.1016/S0006-3495(96)79473-0 PMC1233765 8913616

[10] Kim MJ, Zheng S, Kim TS, Kim SK (2011) Analysis of DNA coverage using enzymatic cleavage of fluorescent labels. BioChip J 5: 39–46 10.1007/s13206-011-5107-2

[11] He Z, Wu L, Li X, Fields MW, Zhou J (2005) Empirical establishment of oligonucleotide probe design criteria. Appl Environ Microbiol 71: 3753–3760 10.1128/AEM.71.7.3753-3760.2005 16000786 PMC1169010

[12] Dugat-Bony E, Peyretaillade E, Parisot N, Biderre-Petit C, Jaziri F, et al (2012) Detecting unkown sequences with DNA microarrays: explorative probe design strategies. Environ Microbiol 14: 356–371 10.1111/j.1462-2920.2011.02559.x 21895914

[13] Safavieh M, Ahmed MU, Sokullu E, Ng A, Braescu L, et al (2014) A simple cassette as point-of-care diagnostic device for naked-eye colorimetric bacteria detection. Analyst 139: 482–487 10.1039/c3an01859h 24300967

[14] Fang X, Chen H, Yu S, Jiang X, Kong J (2011) Predicting viruses accurately by a multiplex microfluidic loop-mediated isothermal amplification chip. Anal Chem 83: 690–695 10.1021/ac102858j 21142070

[15] Andersen D, Nickisch-Rosenegk MV, Bier FF (2009) Helicase-dependent amplification: use in ONChip amplification and potential for point-of-care diagnostics. Expert Rev Mol Diagn 9: 645–650 10.1586/erm.09.46 19817549

[16] Rohrman BA, Richards-Kortum RR (2010) A paper and plastic device for performing recombinase polymerase amplification of HIV DNA. Lab Chip 12: 3082–3088 10.1039/C2LC40423K PMC3569001 22733333

[17] Zanoli LM, Spoto G (2013) Isothermal amplification methods for the detection of nucleic acids in microfluidic devices. Biosensors 3: 18–43 10.3390/bios3010018 25587397 PMC4263587

[18] Craw P, Balachandran W (2012) Isothermal nucleic acid amplification technologies for point-of-care diagnostics: a critical review. Lab on a Chip 12: 2469–2486 10.1039/C2LC40100B 22592150

[19] Kimura Y, de Hoon MJL, Aoki S, Ishizu Y, Kawai Y, et al (2011) Optimization of turn-back primers in isothermal amplification. Nucleic Acids Res Nucl Acids Res 39: e59 10.1093/nar/gkr041 21310714 PMC3089485

[20] PrimerExplorer website. Available: http://primerexplorer.jp/e/v4_manual/pdf/PrimerExplorerV4_Manual_1.pdf. Accessed 2014 May 11.

[21] Mori Y, Notomi T (2009) Loop-mediated isothermal amplification (LAMP): a rapid, accurate, and cost-effective diagnostic method for infectious diseases. J Infect Chemother 15: 62–69. 19396514 10.1007/s10156-009-0669-9 PMC7087713

[22] Zhao YS, Park B, Kreiswirth C, Ginocchio R, Veyret A, et al (2009) Rapid real-time nucleic acid sequence-based amplification-molecular beacon platform to detect fungal and bacterial bloodstream infections. J Clin Microbiol 47: 2067–78. 19403758 10.1128/JCM.02230-08 PMC2708467

[23] Chandler DP, Bryant L, Griesemer SB, Gu R, Knickerbocker AK, et al (2012) Integrated amplification microarrays for Infectious disease diagnostics. Microarrays 1: 107–124 10.3390/microarrays1030107 27605339 PMC5003434

[24] Huang S, Do J, Mahalanabis M, Fan A, Zhao L, et al (2013) Low cost extraction and isothermal amplification of DNA for infectious diarrhea diagnosis. PLoS ONE 8(3): e60059 10.1371/journal.pone.0060059 23555883 PMC3610934

[25] Kamal SAA, Awadh RMJ, Al-Marzoqi AHM (2013) Genetic study of TORCH infections in women with bad obstetric history: multiplex polymerase chain reaction for detection of common pathogens and agents of congenital infections. Journal of Biology, Agriculture and Healthcare 3: 49–53.

