# Supplementary material for: A Novel Cassette Method for Probe Evaluation in the Designed Biochips
Source: PLoS One. 2014 Jun 4;9(6):e98596. doi: 10.1371/journal.pone.0098596 (PMC4045846; doi:10.1371/journal.pone.0098596)
Supplement: Data S1 — The composition and sequences of N1, N2, N3, N4 and N5 cassettes. (DOC) [file pone.0098596.s001.doc]

**DATA S1**

**N1 cassette**: 5'-T7*CT1*CT2*CT3*SP6-3'

**N1 cassette sense**: 5'-GTA ATA CGA CTC ACT ATA GGG*GGA GAT AAT GAG AAC CAT GCT AC*TCT ATG GGA AGG TTT CGG C*TCA CCA CCT ACA CGG AAA CA*CTA TAG TGT CAC CTA AAT CGT A-3'

**N2 cassette**: 5'-T7*CMV1*CMV2*HSV*TOXO1*RV*SP6-3'

**N2 cassette sense**: 5'-GTA ATA CGA CTC ACT ATA GGG*ATA AGC GGG AGA TGT GGA TG*GCG GTG TGC TTT ATT AGG G*TTA TCA ACC GCA CCT CCA G*CCG GAA ATA GAA AGC CAT G*AAC GCC ATT CCC CTG ACT*CTA TAG TGT CAC CTA AAT CGT A-3'

**N3 cassette**: 5'-T7*TOXO1*CMV2*HSV*CMV1*RV*SP6-3'

**N3 cassette sense**: 5'-GTA ATA CGA CTC ACT ATA GGG*CCG GAA ATA GAA AGC CAT G*GCG GTG TGC TTT ATT AGG G*TTA TCA ACC GCA CCT CCA G*ATA AGC GGG AGA TGT GGA TG*AAC GCC ATT CCC CTG ACT*CTA TAG TGT CAC CTA AAT CGT A-3'

**N4 cassette**: 5'-T7*TOXO2*TOXO3*TOXO4*TOXO1*SP6-3'

**N4 cassette sense**: 5'-GTA ATA CGA CTC ACT ATA GGG*GTA TTC GCA GAT TGG TCG C*GGT GAC GAA AGG GGA AGA AT*CCT GTT TCC TCT CTT CAC TGT C*CCG GAA ATA GAA AGC CAT G*CTA TAG TGT CAC CTA AAT CGT A-3'

**N5 cassette**: 5'-T7*CT1*CMV2*HSV*RV*TOXO2*SP6-3'

**N5 cassette sense**: 5'-GTA ATA CGA CTC ACT ATA GGG*GGA GAT AAT GAG AAC CAT GCT AC*GCG GTG TGC TTT ATT AGG G*TTA TCA ACC GCA CCT CCA G *AAC GCC ATT CCC CTG ACT*GTA TTC GCA GAT TGG TCG C*CTA TAG TGT CAC CTA AAT CGT A-3'
